# Cardiac Lymphoma Presenting as Superior Vena Cava Syndrome Mimicking Venous Thrombosis

**DOI:** 10.7759/cureus.109846

**Published:** 2026-05-28

**Authors:** Gayathry Rajasekharan, Parvathy Rajasekharan, Chinthu Sara Jacob, Rajasekharan Chandrasekharan

**Affiliations:** 1 Pathology, Government Medical College, Thrissur, IND; 2 Oncology, PRS Hospital, Trivandrum, IND; 3 Internal Medicine, Sree Uthradom Thirunal (SUT) Academy of Medical Sciences, Trivandrum, IND

**Keywords:** cardiac mass, diffuse large b-cell lymphoma (dlbcl), primary cardiac lymphoma (pcl), right atrial cardiac mass, superior vena cava (svc) syndrome

## Abstract

Primary cardiac lymphoma is an exceptionally rare extranodal non-Hodgkin lymphoma that often presents with non-specific cardiopulmonary symptoms, resulting in delayed diagnosis. Superior vena cava (SVC) syndrome as the initial manifestation is particularly uncommon and may mimic more prevalent conditions such as thrombosis, mediastinal malignancy, or right atrial myxoma. We report a case of a 37-year-old immunocompetent woman who presented with progressive breathlessness, facial puffiness, distended neck veins, and upper limb edema for two weeks. Initial Doppler evaluation suggested internal jugular venous thrombosis with distal obstruction, and anticoagulation therapy was initiated. However, persistent symptoms prompted further evaluation. Transthoracic echocardiography demonstrated a large homogeneous right atrial mass extending into the coronary sinus, while computed tomography revealed a non-enhancing mass occupying the right atrium with extension into the superior vena cava. Cardiac magnetic resonance imaging was inconclusive. Given persistent symptoms and uncertainty regarding the diagnosis, surgical exploration with biopsy was performed. Histopathological examination with immunohistochemistry confirmed diffuse large B-cell lymphoma with CD20 and CD30 positivity, establishing primary cardiac lymphoma.

This case highlights the variable presentation of primary cardiac lymphoma and the limitations of imaging in differentiating malignant cardiac masses from thrombus. Although uncommon, primary cardiac lymphoma should be considered in patients with unexplained SVC syndrome, particularly when anticoagulation fails or imaging demonstrates right atrial involvement. Early tissue diagnosis is essential, as prompt initiation of chemotherapy may improve outcomes in this aggressive yet potentially treatable malignancy.

## Introduction

Primary cardiac tumors are among the rarest neoplasms encountered in clinical practice, with reported incidences ranging from 0.001% to 0.03% in autopsy series. Most lesions are benign, with myxomas being the most common subtype. Primary malignant cardiac tumors are distinctly uncommon, accounting for approximately 15-25% of primary cardiac neoplasms, with sarcomas, particularly angiosarcoma of the right atrium, being the predominant subtype [1]. Primary cardiac lymphoma (PCL) represents an exceptionally rare entity, accounting for nearly 1-2% of primary cardiac tumors and less than 1% of extranodal non-Hodgkin lymphomas [1]. Owing to its rarity, variable presentation, and diagnostic complexity, PCL remains a significant clinical challenge.

Primary cardiac lymphoma is defined as lymphoma involving the heart and/or pericardium, with the principal tumor burden confined to the heart or pericardium at diagnosis. Most reported cases are diffuse large B-cell lymphomas, although other histological variants have been described. The disease occurs more frequently in immunocompromised individuals but may also arise in immunocompetent patients. The right atrium is the most commonly involved chamber, and tumor infiltration may extend into adjacent great vessels, the pericardium, or conduction tissue, resulting in varied clinical manifestations [2].

The clinical presentation of PCL is often non-specific and depends on tumor size, location, and extent of infiltration. Symptoms may include dyspnea, chest discomfort, arrhythmias, syncope, heart failure, or pericardial effusion. Superior vena cava (SVC) syndrome as the presenting manifestation is exceedingly rare and may mimic more common conditions such as bronchogenic carcinoma, mediastinal lymphoma, thrombosis, or fibrosing mediastinitis, frequently delaying diagnosis and treatment [3].

Multimodality imaging, including echocardiography, contrast-enhanced computed tomography, and cardiac magnetic resonance imaging, plays an important role in evaluating intracardiac masses and the extent of disease. However, imaging findings may be non-specific, making histopathological confirmation with immunohistochemistry the diagnostic gold standard [4,5].

Recognition of PCL is clinically important because, unlike many other primary cardiac malignancies, it may respond favorably to systemic chemotherapy, particularly rituximab-based regimens for CD20-positive diffuse large B-cell lymphoma. Early diagnosis may improve survival and relieve hemodynamic compromise. We report a rare case of primary cardiac diffuse large B-cell lymphoma arising in the right atrium with extension into the superior vena cava and internal jugular vein, presenting as superior vena cava syndrome in a young immunocompetent woman. This case highlights the importance of considering cardiac malignancy in patients with unexplained venous obstruction and intracardiac masses [5].

## Case presentation

A 37-year-old female was referred to the internal medicine outpatient department with gradually progressive breathlessness of two weeks' duration, facial puffiness, and edema of the upper extremities. She was apparently normal before she noticed the symptoms and had no history of similar illness in the past. There was no associated history of fever, sore throat, cough, chest pain, expectoration or hemoptysis, paroxysmal nocturnal dyspnea, orthopnea, pruritus, night sweats, loss of appetite, loss of weight, rashes, recurrent abortions, arthritis, oral ulceration, or intake of oral contraceptive pills. On examination, the patient was conscious, well-oriented, and not distressed. Her temperature was 99°F, respiratory rate was 20 breaths per minute, pulse rate was 100 beats per minute, and blood pressure in the right upper limb, supine position, was 110/70 mmHg. The face was puffy, and there were marked dilated non-pulsatile veins in the neck. There was no evidence of pallor, cyanosis, finger clubbing, lymphadenopathy, or pitting pedal edema. Examination of the cardiovascular system revealed normal heart sounds, no additional sounds or murmurs. Examinations of the gastrointestinal system and neurological system were within normal limits. The results of the investigations on the day of admission are summarized in Table 1.

**Table 1:** Initial laboratory investigations demonstrated predominantly normal parameters, with elevated erythrocyte sedimentation rate and mild hypoalbuminemia. AST: aspartate aminotransferase; ALT: alanine aminotransferase; INR: international normalized ratio; PT: prothrombin time; aPTT: activated partial thromboplastin time; FDP: fibrin degradation products

Parameters	Values	Reference range
Hemoglobin	112 g/L	120-150 g/L
Total WBC count	8.9x10^9^/L	4.0-11.0x10^9^/L
Neutrophils	75%	40%-75%
Lymphocytes	20%	20%-45%
Eosinophils	5%	1%-6%
Platelet count	185x10^9^/L	150-450x10^9^/L
ESR at the end of the first hour	60 mm/h	0-20 mm/h
Blood urea	10.7 mmol/L	2.5-7.1 mmol/L
Random blood sugar	5.3 mmol/L	3.9-7.8 mmol/L
Sodium	136 mmol/L	135-145 mmol/L
Potassium	4.8 mmol/L	3.5-5.1 mmol/L
Total bilirubin	22.2 µmol/L	5-22 µmol/L
AST	84 U/L	5-40 U/L
ALT	28 U/L	7-56 U/L
Alkaline phosphatase	140 U/L	44-147 U/L
Total protein	72 g/L	60-83 g/L
Serum albumin	30 g/L	35-50 g/L
PT-INR	1.1	0.8-1.2
aPTT	26.1 s	25-35 s
D-dimer	220 ng/mL	<500 ng/mL
Urine FDP	6 µg/mL	<10 µg/mL

Electrocardiogram revealed sinus tachycardia with no ST-T changes. Work-up for immunocompromised status included a complete blood count with differential, renal and liver function tests, blood glucose/HbA1c, HIV testing, serum immunoglobulin levels, CD4/CD8 counts, and evaluation for malignancy, autoimmune disorders, or immunosuppressive drug use, based on clinical suspicion. ANA and dsDNA were also negative. Epstein-Barr virus (EBV) VCA IgM and Cytomegalovirus (CMV) IgM were negative. HBsAg and hepatitis B virus (HBV) and hepatitis C virus (HCV) tests were negative. Bone marrow aspiration and trephine biopsy findings were normal. Doppler study of the neck veins revealed a dilated internal jugular vein with streamlined flow, indicating distal obstruction (Figure 1, panel A). End-on view of the internal jugular vein (IJV) showed thrombus in its lumen (Figure 1, panel B).

**Figure 1:**
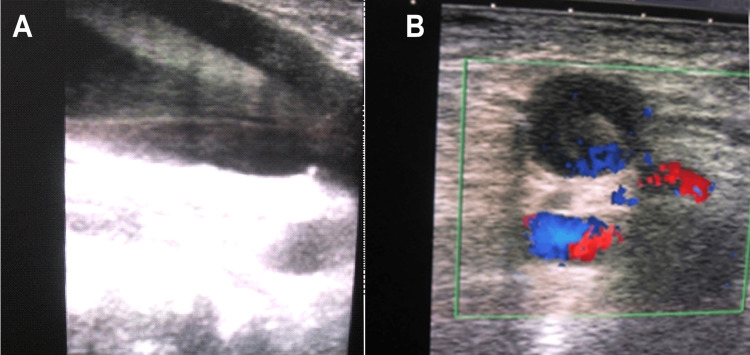
Doppler study of neck veins. Doppler study of the neck vein showed a dilated internal jugular vein with streamlined flow, indicating distal obstruction (A). An end-on view of IJV showed thrombus within its lumen (B). IJV: internal jugular vein

Transthoracic echocardiography (apical four-chamber view) with color Doppler showed a dilated right atrium with a homogeneous mass measuring 4.6x3.7 cm extending into the coronary sinus, no gradient across the tricuspid valve, trivial tricuspid regurgitation, a small inter-atrial septal defect with continuous flow across the septum, and a thin rim of pericardial effusion (Figure 2).

**Figure 2:**
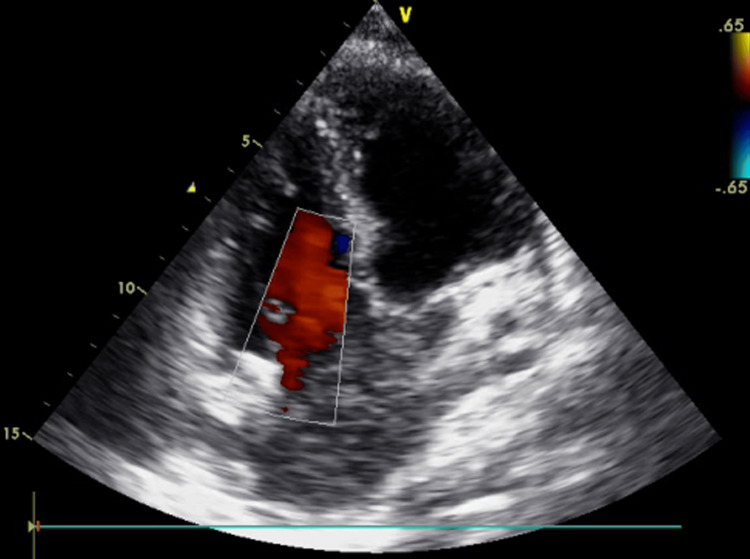
Transthoracic echocardiography (apical four-chamber view) with color Doppler study. A transthoracic echocardiography (apical four-chamber view) with color Doppler revealed a dilated right atrium with a homogeneous mass measuring 4.6x3.7 cm extending into the coronary sinus, no gradient across the tricuspid valve, trivial tricuspid regurgitation, a small inter-atrial septal defect with continuous flow across the septum, and a thin rim of pericardial effusion.

CT chest revealed a homogeneous non-contrast-enhancing mass involving the entire chamber of the right atrium, extending to the lumen of the superior vena cava and projecting to the left atrium through the small atrial septal defect, and a dilated right atrium (Figure 3, panels A, B). An MRI heart showed a homogeneous mass involving the entire chamber of the right atrium, extending to the lumen of the superior vena cava and projecting to the left atrium through the small atrial septal defect and a dilated right atrium (Figure 3, panel C). PET-CT was advised, but could not be done due to financial constraints.

**Figure 3:**
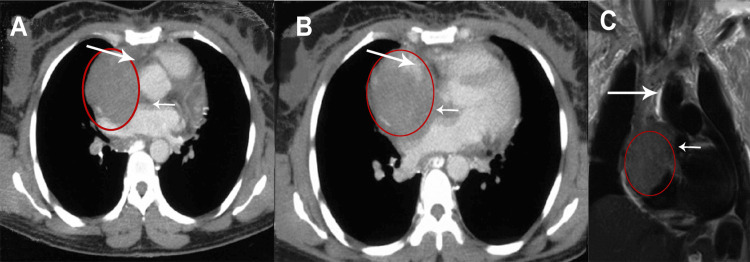
CT chest and MRI heart. Chest CT demonstrated a homogeneous non-contrast-enhancing mass involving the entire chamber of the right atrium (red circle), extending into the lumen of the superior vena cava (white arrows above) and projecting into the left atrium through the small atrial septal defect (lower white arrow), with dilation of the right atrium (A, B). Cardiac MRI showed a homogeneous mass involving the entire chamber of the right atrium (red circle), extending into the lumen of the superior vena cava (white arrows above) and projecting into the left atrium through the small atrial septal defect (lower white arrow), with dilation of the right atrium (C).

Intraoperative images showing an intracardiac tumor seen in the right atrium with extension into the superior vena cava and innominate vein, with superior vena cava obstruction and infiltration into the right atrial wall, right atrioventricular groove, and the space between the aorta and superior vena cava (Figure 4, panels A-C).

**Figure 4:**
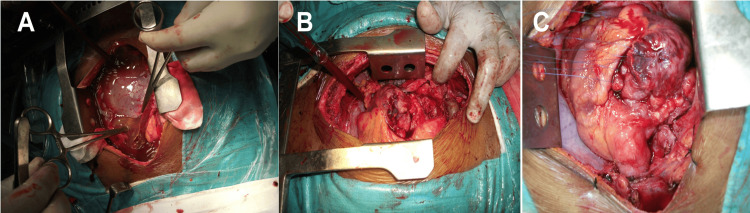
Intraoperative photographs showing a right atrial mass and its extent (A, B, and C). Intraoperative images showing an intracardiac tumor seen in the right atrium with extension into the superior vena cava and innominate vein, with superior vena cava obstruction and infiltration into the right atrial wall, right atrioventricular groove, and the space between the aorta and superior vena cava.

A low-power view of the biopsy specimen showed sheets of atypical lymphoid cells distributed in the cardiac tissue (Figure 5, panel A). High-power view of the biopsy specimen showed pleomorphic cells with scant cytoplasm, a high nuclear-to-cytoplasmic ratio, hyperchromatic nuclei with indistinct nucleoli, and visible mitotic figures (Figure 5, panel B).

**Figure 5:**
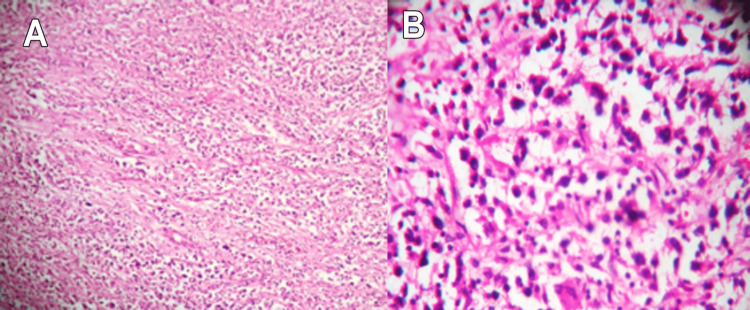
Biopsy specimen low power and high power view. A low-power view of the biopsy specimen showed sheets of atypical lymphoid cells distributed in the cardiac tissue (A). A high-power view of the biopsy specimen showed pleomorphic cells with scant cytoplasm, a high nuclear-to-cytoplasmic ratio, hyperchromatic nuclei with indistinct nucleoli, and visible mitotic figures (B).

Immunohistochemistry revealed neoplastic cells negative for CD3 (Figure 6, panel A), with diffuse strong membranous positivity for CD20 (Figure 6, panel B) and CD30 positivity (Figure 6, panel C). This confirmed the diagnosis of primary cardiac lymphoma, non-Hodgkin lymphoma.

**Figure 6:**
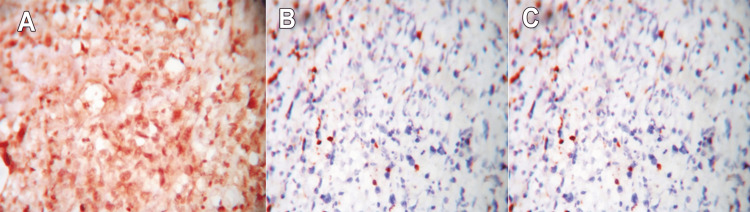
Immunohistochemistry confirming the diagnosis of large B-cell lymphoma. Neoplastic cells were negative for CD3 (A), with diffuse strong membranous positivity for CD20 (B) and CD30 positivity (C), confirming the diagnosis of large B-cell lymphoma.

She received anticoagulants and diuretics on admission. She was given two cycles of R-CHOP (rituximab, cyclophosphamide, hydroxydaunorubicin, Oncovin, and prednisolone) every 21 days, but after the second cycle, the patient developed progressive cardiovascular compromise and died.

## Discussion

Primary hematolymphoid tumors of the heart are extremely rare, accounting for nearly 1% of cardiac neoplasms, with primary cardiac lymphomas (PCL) representing only 0.5% of cases [1]. Although immunosuppression, including HIV infection, transplantation, or immunosuppressive therapy, is a recognized risk factor, PCL more commonly occurs in immunocompetent adults, predominantly males aged 55-65 years [3]. However, rare occurrences in young immunocompetent women may contribute to delayed diagnosis [6]. In middle-aged females presenting with SVC syndrome, differential diagnoses include primary or metastatic cardiac tumors, mediastinal malignancies, SVC thrombosis, and mediastinal lymphomas, making PCL an uncommon initial consideration [7,8].

PCL may involve any cardiac chamber or septum but most commonly affects the right side of the heart, especially the right atrium, with epicardial and pericardial extension occurring in nearly 50% of cases [1,9]. According to the 2021 WHO classification, approximately 75% of cardiac tumors are benign and 25% malignant, either primary or metastatic [9,10]. Differential diagnoses of cardiac masses include thrombi, infective lesions such as bacterial or fungal endocarditis, myocardial abscesses, neoplasms, and structural abnormalities such as false tendons and ruptured chordae tendineae [11].

Metastatic cardiac malignancies are 20-40 times more common than primary tumors, with melanoma, lung, breast, and esophageal cancers frequently metastasizing to the heart [12]. Rahouma et al. reported a prevalence of primary malignant cardiac tumors of 10.83%, with angiosarcoma, leiomyosarcoma, and diffuse large B-cell lymphoma (DLBCL) being the most common subtypes [13]. PCL accounts for only 1% of primary cardiac malignancies and 0.5% of extranodal lymphomas [1].

The most frequent sites of involvement include the right atrium (66-77%), right ventricle (25-37%), left atrium (12-30%), left ventricle (2-21%), and septum (2-41%), while pericardial involvement is less common [1,14]. Histologically, DLBCL is the predominant subtype (71-92%), followed by Burkitt lymphoma, chronic lymphocytic leukemia/small lymphocytic lymphoma (CLL/SLL), T-cell lymphomas, and other uncommon variants [1,14]. Cardiac lymphomas are more frequent in immunocompromised individuals, and HIV infection increases the risk of systemic non-Hodgkin lymphoma by 60-200 times compared with the general population [15].

Echocardiography is the initial non-invasive modality that provides real-time assessment of tumor size, morphology, mobility, location, and relationship to adjacent structures, and can also detect hypoechoic masses and pericardial effusion [12]. Although transthoracic echocardiography (TTE) is commonly used, transesophageal echocardiography (TEE) offers superior visualization of deeper cardiac structures and improves diagnostic accuracy [14]. Cardiac CT and MRI help determine the extent of disease and guide biopsy or surgical planning [11]. Positron emission tomography (PET) may identify extracardiac disease or occult primary tumors. Definitive diagnosis requires histopathological examination of endomyocardial biopsy, though cytological analysis of pericardial fluid may aid diagnosis in cases with effusion [10,15,16].

Most PCLs are non-Hodgkin lymphomas, predominantly DLBCL, which accounts for over 80% of cases [1,10]. Rare subtypes include Burkitt lymphoma, T-cell lymphoma, fibrin-associated DLBCL (FA-DLBCL), and primary effusion lymphoma involving the pericardium [1,10]. Cardiac DLBCL is often associated with impaired immunity and includes EBV-associated DLBCL, DLBCL related to chronic inflammation, and FA-DLBCL [17]. Histologically, DLBCL demonstrates large dyscohesive cells with cellular monotony, while immunophenotyping is essential to determine the cell of origin, MYC/BCL2 co-expression, and Epstein-Barr virus (EBV) status [1,16]. FA-DLBCL is usually found incidentally in chronic fibrin deposits, pseudocysts, or hematomas without tissue infiltration [17].

Molecular studies using fluorescence in situ hybridization (FISH) and polymerase chain reaction (PCR) have identified MYC, BCL2, and BCL6 rearrangements in subsets of cardiac DLBCL [16]. Double-hit lymphomas involving MYC and BCL2 rearrangements exhibit aggressive behavior due to increased proliferation and apoptotic resistance, leading to rapid progression and poor chemotherapy response [18]. Such tumors are associated with reduced survival, even when initially confined to the heart [14]. In our patient, immunohistochemistry showed CD20 and CD30 positivity, and the rapid deterioration despite R-CHOP therapy suggests a possible double-hit mechanism.

Chemotherapy remains the mainstay of treatment, particularly the R-CHOP regimen (rituximab, cyclophosphamide, hydroxydaunorubicin, Oncovin, and prednisolone), with response rates of 79-87% [19]. Early treatment may result in rapid symptomatic improvement and complete remission. However, extensive tumor infiltration into the SVC and internal jugular vein in our patient likely contributed to rapid cardiovascular compromise and death after only two treatment cycles. Radiotherapy may be useful in refractory or residual disease [16,19]. Surgery is generally reserved for urgent debulking in patients with severe complications such as SVC syndrome or worsening heart failure [14,19]. Prognosis has improved compared with earlier perceptions, with remission rates approaching 60% after chemotherapy alone [12,16]. Novel therapeutic approaches that combine chimeric antigen receptor T-cell (CAR-T) therapy with autologous stem cell transplantation (ASCT) have achieved sustained remissions in selected patients [18]. CAR-T therapy is currently recommended in relapsed (<12 months) or refractory disease.

## Conclusions

PCL is a rare but potentially treatable malignancy with non-specific clinical features that often delay diagnosis. This case highlights an unusual presentation with SVC syndrome in a young immunocompetent woman initially mistaken for venous thrombosis. Persistent symptoms despite anticoagulation and detection of a right atrial mass necessitated further evaluation. While multimodality imaging aids diagnosis and disease assessment, definitive diagnosis depends on histopathology with immunohistochemistry. Early recognition and prompt chemotherapy are essential for improving outcomes. Clinicians should maintain suspicion for PCL in unexplained SVC obstruction or right atrial masses, even outside the typical demographic profile. Despite therapeutic advances, advanced PCL remains aggressive with a poor prognosis, as demonstrated in this case.
